# Islet Lymphocytes Maintain a Stable Regulatory Phenotype Under Homeostatic Conditions and Metabolic Stress

**DOI:** 10.3389/fimmu.2022.814203

**Published:** 2022-01-25

**Authors:** Jennifer C. Whitesell, Robin S. Lindsay, Jessica G. Olivas-Corral, Seth F. Yannacone, Mary H. Schoenbach, Erin D. Lucas, Rachel S. Friedman

**Affiliations:** ^1^ Department of Immunology and Microbiology, University of Colorado Anschutz Medical Campus, Aurora, CO, United States; ^2^ Barbara Davis Center for Diabetes, Aurora, CO, United States; ^3^ Department of Immunology and Genomic Medicine, National Jewish Health, Denver, CO, United States

**Keywords:** islet, T cell, regulatory B cell, resident lymphocyte, type 2 diabetes, type 1 diabetes, tissue homeostasis

## Abstract

T cells and B cells have been identified in human and murine islets, but the phenotype and role of islet lymphocytes is unknown. Resident immune populations set the stage for responses to inflammation in the islets during homeostasis and diabetes. Thus, we sought to identify the phenotype and effector function of islet lymphocytes to better understand their role in normal islets and in islets under metabolic stress. Lymphocytes were located in the islet parenchyma, and were comprised of a mix of naïve, activated, and memory T cell and B cell subsets, with an enrichment for regulatory B cell subsets. Use of a Nur77 reporter indicated that CD8 T cells and B cells both received local antigen stimulus, indicating that they responded to antigens present in the islets. Analysis of effector function showed that islet T cells and B cells produced the regulatory cytokine IL-10. The regulatory phenotype of islet T cells and B cells and their response to local antigenic stimuli remained stable under conditions of metabolic stress in the diet induced obesity (DIO) model. T cells present in human islets retained a similar activated and memory phenotype in non-diabetic and T2D donors. Under steady-state conditions, islet T cells and B cells have a regulatory phenotype, and thus may play a protective role in maintaining tissue homeostasis.

## Introduction

There is increasing evidence that islet inflammation not only occurs in autoimmune type 1 diabetes (T1D), but also in type 2 diabetes (T2D) (1, 2). In T2D, insulin resistance combined with increased demand for insulin results in beta cell dysfunction and hyperglycemia (3, 4). T2D islets also contain an increased number of macrophages compared to non-diabetic islets (2, 5). Obesity is a common comorbidity with T2D patients (6), which can be associated with adipose tissue inflammation that results in increased circulating pro-inflammatory cytokines such as TNFα and IL-6 (6, 7). Because islets are highly vascularized, the islet environment is sensitive to circulating and local cytokines. In T2D, islet macrophages convert from an anti-inflammatory M2 state to M1-like phenotype and produce TNFα (8, 9). Hyperglycemia and circulating leptin trigger further production of pro-inflammatory cytokines, such as IL-1β, within the islet (10). IL-1β and hyperglycemia may trigger apoptosis of beta cells, further contributing to the development of T2D (1, 7, 11–13).

In addition to macrophages, T cells and B cells have been identified in non-diabetic human and mouse islets (14–18). In human islets, 80% of the T cells were memory CD8 T cells, with most of the cells also expressing CD103 (14). In murine islets, the T cell compartment includes CD4+, CD8+ and regulatory T cells which increase with age (18). B cells have also been identified in human and mouse islets at low numbers (14–18). It is unclear what function islet T cells and B cells may be performing and how these populations change when the islet environment is altered by inflammation.

Regulating the balance between pro- and anti-inflammatory cytokines is important in maintaining tissue homeostasis. This is especially important in the highly vascularized islet environment due to its exposure to circulating cytokines. IL-10 is a regulatory cytokine which functions to resolve inflammation, promote tissue repair, maintain tissue homeostasis (19, 20) and control production of proinflammatory cytokine IFN-γ (21). Expression of IL-10 in the islets can protect islets by reducing IL-1β-induced Fas-ligand expression in beta cells leading to reduced beta cell apoptosis (22). However in T2D, hyperglycemia induces islet macrophages to become less responsive to IL-10 signaling and to maintain a more pro-inflammatory M1-like state (23).

Tissue resident lymphocytes can assist in promoting tissue homeostasis in many peripheral tissues including the pancreas (14, 24–27). Tissue resident memory T cells (Trms) are thought to serve as sentinels alongside the resident myeloid compartment. Most Trms display a “memory”-like surface phenotype and may express CD69 which prevents tissue egress and/or CD103 which promotes cell adhesion within a tissue (27–29). Trms are poised to rapidly produce proinflammatory cytokines and cytolytic molecules when the tissue becomes damaged (27, 30, 31). Trms across tissues have a distinct transcriptional profile which includes expression of IL-2, IFN-γ, and IL-10 (31, 32). Production of IL-10 under steady-state conditions by Trms may promote tissue homeostasis (31).

There is a gap in knowledge about the phenotype and function of islet immune cell populations under homeostatic conditions. Understanding the role of islet lymphocytes at baseline is key to understanding how immune cells promote or hinder the development of diabetes.

In this paper, we sought to characterize islet T cell and B cell populations under homeostatic conditions and under metabolic stress in a murine diet-induced obesity (DIO) model (33, 34). Using 2-photon imaging and fluorescent reporter mice on the non-diabetic C57BL/6 background, we found that islet T cells and B cells were present in the parenchyma of the islet, received local antigen receptor signaling, and had a mixed naïve and activated phenotype. Like murine islets, human non-diabetic and T2D islet T cells contained a mix of naïve and activated/memory cells. The islet lymphocytes expressed the regulatory cytokine IL-10, but not the pro-inflammatory cytokine IFN-γ, suggesting that they play a regulatory role. Notably, islet T cells and B cells maintained their regulatory phenotype in the context of systemic inflammation and metabolic stress in the DIO model. These data indicate that islet T cells and B cells may have a regulatory role in the islets during homeostasis and in T2D.

## Results

### T Cells and B Cells Are Present in the Pancreatic Islet Parenchyma of Autoimmune Resistant Mice

**Figure 1:**
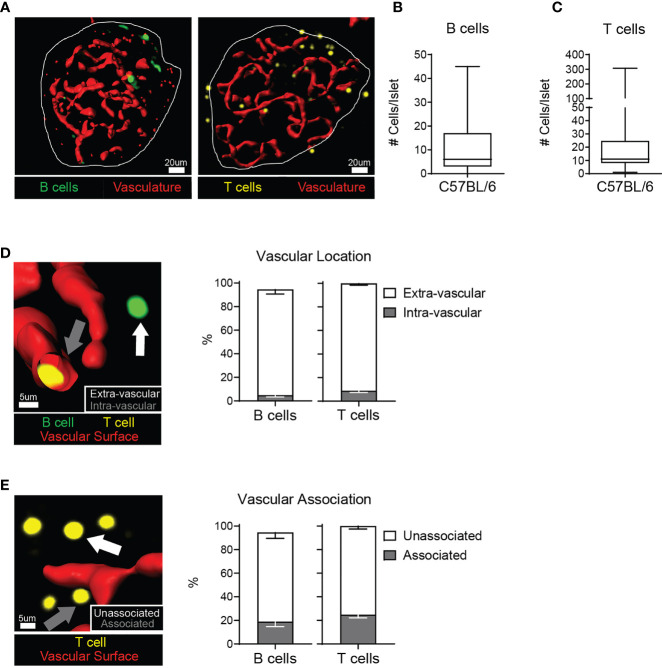
C57BL/6 islets contain T cells and B cells in the parenchyma. Whole explanted C57BL/6 islets were stained for the vasculature (anti-CD31; red), T cells (anti-CD3; yellow), and B cells (anti-B220; green) and imaged by 2-photon microscopy. **(A)** Representative images. Vasculature is represented by a 3D surface rendering. **(B)** Number of B220+ cells per islet. **(C)** Number of CD3+ cells per islet. **(D)** Example image and quantification of the intra- or extra-vascular location of T cells and B cells. White arrow: extra-vascular cell. Grey arrow: intra-vascular cell. **(E)** Example image and quantification of the vascular association of extra-vascular T cells and B cells. White arrow: Unassociated cell - greater than 1 μm away from the vasculature surface. Grey arrow: Associated cell – within 1 μm of the vascular surface. **(A–E)** n=37 islets from 3 independent experiments for B cells. n=72 islets from 5 independent experiments for T cells. Error bars: SEM.

The localization of an immune cell within a tissue can shed light on its function. Immune cells can pass through tissues in the vasculature, or migrate out of the vasculature to patrol the parenchyma. We analyzed lymphocyte localization in the islets of 12-20 week old C57BL/6 (B6) mice, because of their resistance to autoimmunity (35) and propensity for glucose intolerance (36). We stained isolated whole islets, and visualized the T cells, B cells, and vasculature by 2-photon microscopy. T cells and B cells were present in non-diabetic murine islets (**Figures 1A–C**). We next determined that T cells and B cells were primarily located in the islet parenchyma by generating a three-dimensional vascular surface based on CD31 staining and classifying the T cell and B cell localization relative to the vasculature (**Figures 1D, E**).

### C57BL/6 Islets Contain a Mix of Naïve, Activated, and Resident Memory CD4 and CD8 T Cells

**Figure 2:**
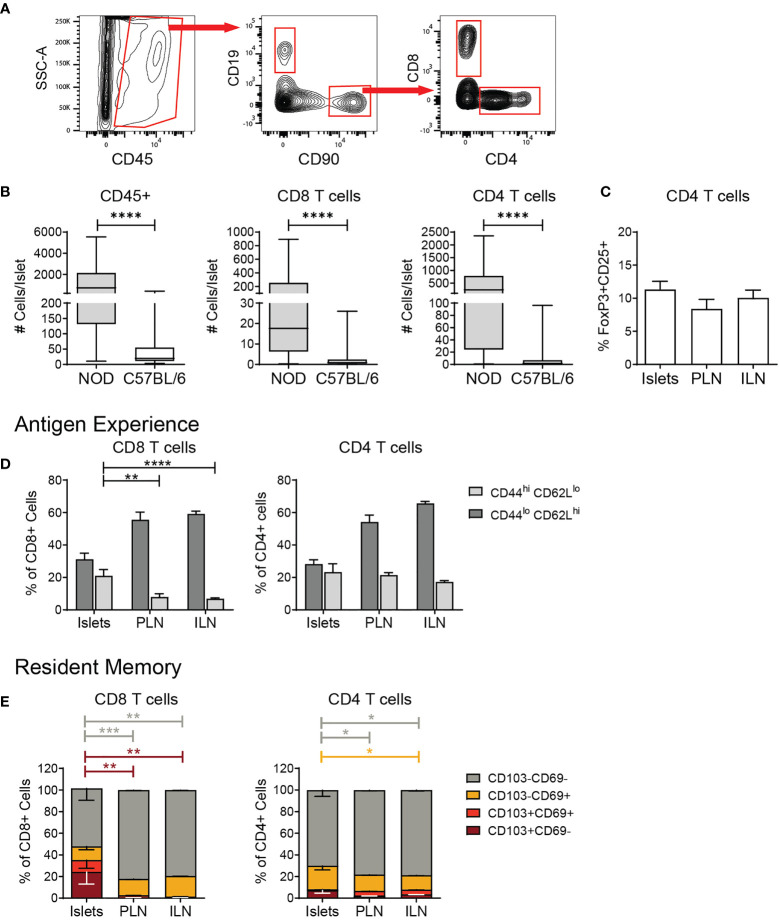
Islet T cells are comprised of naïve, activated, and resident memory CD4 and CD8 T cells. Flow cytometry analysis of T cells in islets and lymph nodes. **(A)** Example gating scheme of islet lymphocytes. **(B)** Number CD45+ cells, CD8+ T cells, and CD4+ T cells per islet. n=49-57 C57BL/6 mice from 8-10 independent experiments, n=6 NOD mice from 5 independent experiments. **(C)** Percent FoxP3+CD25+ cells of CD4+ T cells. n=9 C57BL/6 mice from 3 independent experiments. **(D)** Quantification of CD44 expression in T cells. **(E)** Percent CD103 and/or CD69 expressing T cells. n=8 C57BL/6 mice from 2 independent experiments. **(A–E)** Statistics: *p < 0.05, **p < 0.01, ***p < 0.001, ****p < 0.0001. Error bars: SEM. **(B)** Mann Whitney two-tailed test. **(C)** One way ANOVA with Tukey’s multiple comparisons test. **(D, E)** Two-way ANOVA with Tukey’s multiple comparisons test.

Next, we determined the phenotype of the islet T cells. As a benchmark of T cell numbers in the islets, we compared B6 islets to non-obese diabetic (NOD) islets with autoimmune insulitis. Single-cell islet suspensions were stained with fluorescent antibodies for flow cytometry analysis, and gated on lymphocyte subsets (**Figure 2A**). T cell numbers in B6 islets were significantly lower than in inflamed NOD islets (**Figure 2B**). CD4^+^CD25^+^FoxP3^+^ regulatory T cells (Tregs) were present in B6 islets at a similar frequency as lymph nodes (**Figure 2C**). Approximately one third of the CD4 and CD8 T cells present in B6 islets had a naïve CD44^lo^CD62L^hi^ phenotype (**Figure 2D**). Activated CD44^hi^CD62L^lo^ CD8 T cells were enriched in B6 islets compared to draining and non-draining lymph nodes (**Figure 2D**). In contrast, the frequency of activated CD4 T cells in B6 islets was similar to the lymph nodes (**Figure 2D**).

We next asked if B6 islet T cells had a tissue resident, Trm-like phenotype by flow cytometry analysis of CD103 and CD69 expression. While islet CD8 T cells showed an enrichment for Trms with a higher percentage of CD103+CD69- cells in comparison to the lymph nodes, these populations in islet CD4 T cells were similar to lymph nodes (**Figure 2E**). Overall, these data show that the T cell compartment of normal B6 islets is comprised of a mix of naïve, activated, and memory, effector and regulatory CD4 and CD8 T cells.

### Human T2D Islets Contain Activated and Memory T Cells

**Figure 3:**
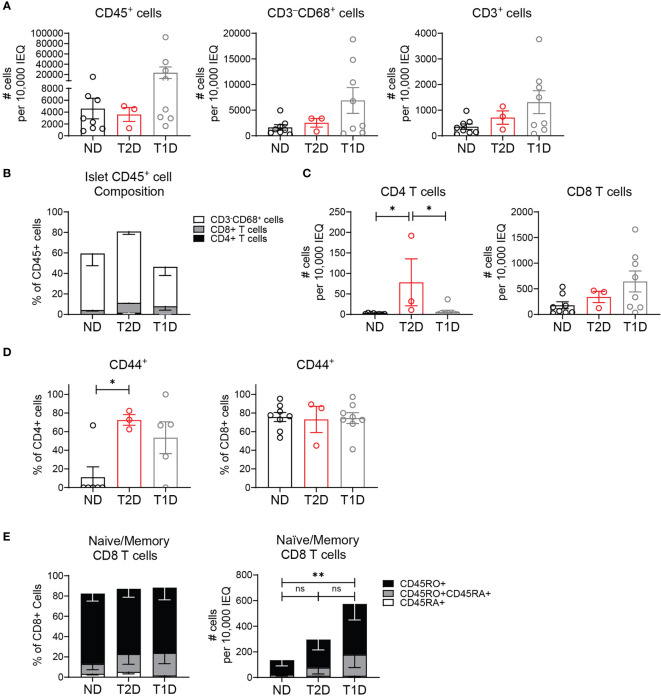
Human T2D islets contain activated and memory T cells. HPAP non-diabetic (ND), type 2 diabetic (T2D), and type 1 diabetic (T1D) islets analyzed by CyTOF. **(A)** Number CD45+ cells, CD3-CD68+ cells, and CD3+ T cells per 10,000 IEQ. **(B)** Percentage CD3-CD68+ cells, CD3+CD4+ T cells and CD3+CD8+ T cells of islet CD45+ population. **(C)** Number CD3+CD4+ T cells and CD3+CD8+ T cells per 10,000 IEQ. **(D)** Quantification of CD44 expression in islet T cells. **(E)** Quantification of CD45RA (naïve), CD45RO+CD45RA+ (activated and transitioning to memory) and CD45RO (memory) expression in islet CD8 T cells. **(A–E)** n=8 control non-diabetic donors, 3 T2D donors, 8 T1D donors. Statistics: *p < 0.05, **p < 0.01. Error bars: SEM. One-way ANOVA with Tukey’s multiple comparison test.

We next asked if non-diabetic human islets contained T cells as we observed in murine islets. We analyzed whether the islet T cells were altered in T2D, since T2D islets become inflamed by pro-inflammatory macrophages which could drive recruitment of T cells to the islets (1, 2, 8, 9). We also compared the T cells in T2D islets to those in T1D islets. To do this, 3 T2D donors, 8 T1D donors, and 8 non-diabetic control donors without islet autoantibodies were selected from the Human Pancreas Analysis Program (HPAP) Database consortia under Human Islet Research Network (RRID : SCR_014393) (37). We analyzed CyTOF data of islet samples and found a similar number of CD45+ cells between T2D and non-diabetic donors (**Figure 3A**). Of CD45+ cells, there was no significant difference in the frequency of CD4 T cells, CD8 T cells and CD68+ myeloid cells between T1D, T2D and non-diabetic control islets (**Figure 3B**). T2D and non-diabetic donors also had similar numbers of CD3+ T cells (**Figure 3A**). Interestingly, T2D donor islets had a significantly higher number of CD4 T cells compared to non-diabetic and T1D islets but had similar numbers of CD8 T cells (**Figure 3C**).

Analysis of islet T cell activation state by CD44 expression showed that islet CD8 T cells had a largely activated or memory phenotype, and the activation state was not significantly different between T2D, T1D, and control donors (**Figure 3D**). However, islet CD4 T cell activation was significantly higher in T2D donors compared to non-diabetic donors but similar to T1D donors (**Figure 3D**). In the islet CD8 T cell population, there was no significant difference in the frequency of naïve (CD45RA+) T cells, memory (CD45RO+) T cells, or activated T cells transitioning to memory (CD45RO+CDRA+) between the T2D, T1D and control donors (**Figure 3E**). As expected, T1D donor samples contained a higher number of memory (CD45RO+) and activated (CD45RO+CD45RA+) CD8 T cells (**Figure 3E**). However, the memory state of T2D islet CD8 T cells was not statistically different from the CD8 T cells in non-diabetic or T1D donor islets. We were unable to assess CD45RA/RO expression in CD4 T cells due their low numbers in the non-diabetic and T1D islet samples. These data show that human islets contain T cell populations with largely activated and memory T cell phenotype in the islets of both T1D and T2D donors.

### Regulatory B Cell Subsets Are Enriched in the Islets

**Figure 4:**
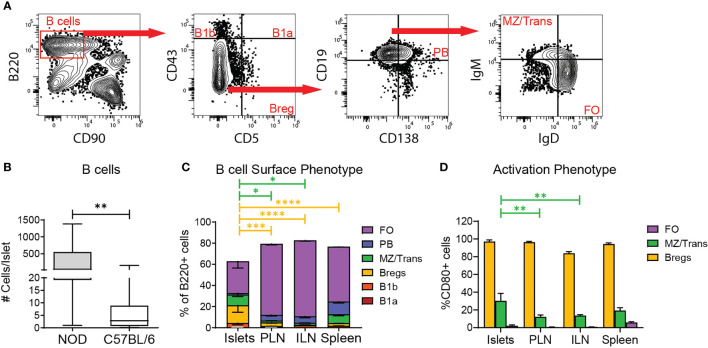
Regulatory B cell subsets are enriched in the islets. **(A)** Example gating of B cells subsets by flow cytometry. **(B)** Number of B cells per islet. n=57 C57BL/6 mice from 10 independent experiments, n=6 NOD mice from 5 independent experiments. **(C)** B cell subset composition of B220+ cells by surface phenotype. FO= CD43-CD5-CD19+CD138-IgM^low^IgD^hi^; PB= CD43-CD5-CD19+CD138+; MZ/Trans= CD43-CD5-CD19+CD138-IgM^hi^IgD^low^; Bregs= CD43-CD5+; B1b= CD43+CD5-; B1a= CD43+CD5+. **(D)** CD80 expression on selected B cell subsets. n=8 C57BL/6 mice from 2 independent experiments. **(B–D)** Statistics: *p < 0.05, **p < 0.01, ***p < 0.001, ****p < 0.0001. Error bars: SEM. **(B)** Mann Whitney two-tailed test. **(C, D)** Two-way ANOVA with Tukey’s Post Test.

Because B cells were found in the parenchyma of murine islets (**Figure 1**) and have been identified within human islets (15–17), we investigated islet B cell phenotype by flow cytometry. Using spleen as a gating control, we classified B220+ B cells as follows: CD43+CD5+ B1a cells (B1a), CD43+CD5- B1b cells (B1b), CD43-CD5+ regulatory B cells (Breg), CD43-CD5-CD19+CD138+ plasma cells and plasmablasts (PB), CD43-CD5-CD19+CD138-IgM^hi^IgD^low^ marginal zone and transitional B cells (MZ/Transitional), and CD43-CD5-CD19+CD138-IgM^low^IgD^hi^ follicular B cells (FO) (**Figure 4A**). B6 islets contained lower numbers of islet B cells than inflamed NOD islets (**Figure 4B**). Islet B cells were composed of multiple subsets with an enrichment for Breg-like B cells and marginal zone/transitional B cells compared to the lymph nodes and spleen (**Figure 4C**). Because murine marginal zone B cells are located exclusively in the spleen, the MZ/Transitional subset in the islets are likely limited to transitional B cells which serve a regulatory function (38). The percentage of B1a and B1b cells in the islets was similar across the tissues (**Figure 4C**).

We next assessed if the major islet B cell subsets had an activated/memory phenotype by CD80 expression. As expected, islet Breg-like cells were largely CD80 positive across tissues, further confirming this subset to be Bregs (**Figure 4D**). Conversely, the MZ/transitional and Follicular B cells were largely naïve due to lack of CD80. These data suggest that the islet B cell population is biased towards a regulatory phenotype based on the enriched frequency of Bregs and transitional B cells, but also contains a significant proportion of naïve B cells.

### Activated CD8 T Cells and B Cells Receive Local Antigen Stimulus in the Islets

**Figure 5:**
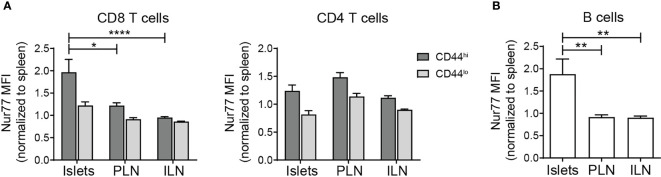
Islet T cells and B cells receive local antigen stimulus. Flow cytometry analysis of C57/BL6.Nur77-GFP mice. Nur77-GFP mean fluorescence intensity (MFI) was normalized to the same lymphocyte subset in the spleen to normalize between experiments. **(A)** Normalized Nur77-GFP expression in T cell subsets. **(B)** Normalized Nur77-GFP expression in B cells. **(A, B)** n=11 mice from 3 independent experiments. Statistics: *p < 0.05, **p < 0.01, ****p < 0.0001. Error bars: SEM. One way ANOVA with Sidak’s multiple comparisons test.

To begin to understand the function of lymphocytes in non-diabetic islets, we analyzed T cell receptor (TCR) and B cell receptor (BCR) signaling. Nur77 expression level serves as a reporter of antigen receptor signaling strength (39, 40). In Nur77-GFP mice, GFP levels correlate with the strength of antigen receptor signaling (39). Thus, to determine if islet T cell and B cell populations received a local antigen stimulus, islets from B6.Nur77-GFP reporter mice were analyzed by flow cytometry. Because baseline Nur77 levels are dependent on T cell activation state, we subsetted the T cells by CD44 expression. The Nur77 signal in T cell and B cell populations were normalized to the same subset in the spleen (**Figures 5A, B**). CD44^hi^ CD8 T cells and B cells expressed higher levels of Nur77-GFP than the same population in the draining and non-draining lymph nodes (**Figures 5A, B**). In contrast, CD4 T cells showed no difference in Nur77 between the islets and lymph nodes (**Figure 5A**). These data suggest that under homeostatic conditions, activated CD8 T cells and B cells receive a local antigen stimulus in the islets.

### Islet T Cells and B Cells Feature A Regulatory Phenotype by Cytokine Production

**Figure 6:**
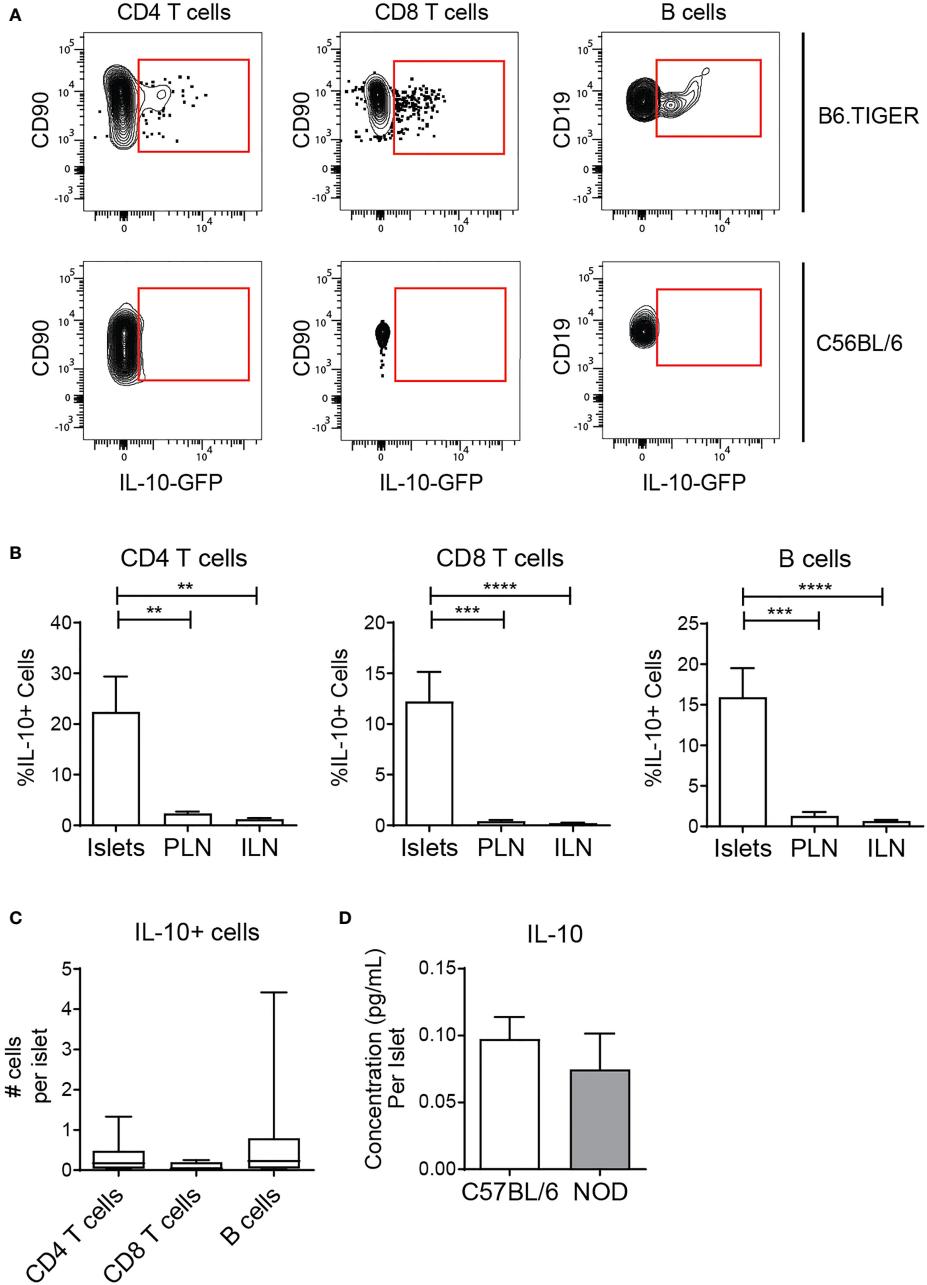
Islet T cells and B cells express the anti-inflammatory cytokine IL-10. **(A, B)** Flow cytometry analysis of IL-10 expression in TIGER IL-10-GFP reporter mice. n=12 mice from 3 independent experiments. **(A)** Representative IL-10-GFP expression in islets. **(B)** Percent IL-10-GFP reporter positive CD4 T cells, CD8 T cells, and B cells. **(C)** Number IL-10-GFP+ CD4 T cells, CD8 T cells, and B cells per islet. **(D)** Secreted IL-10 protein from whole islet cultures. **(B–D)** n=7 C57BL/6 mice, n=6 NOD mice from 3 independent experiments. Statistics: **p < 0.01, ***p < 0.001, ****p < 0.0001. Error bars: SEM. **(B, C)** One way ANOVA with Bonferroni post-test. **(D)** Mann Whitney two-tailed test.

The combination of islet regulatory B cell subsets coupled with local antigen stimulus of T cells and B cells led us to hypothesize that islet lymphocytes function to maintain tissue homeostasis. To test this, we analyzed the regulatory cytokine IL-10 by flow cytometry using B6.TIGER IL-10 reporter mice which express GFP under the IL-10 promoter (41). Islet CD8 T cells, CD4 T cells, and B cells expressed IL-10 transcripts at a higher frequency than the same cell types in the lymph nodes (**Figures 6A, B**). Small numbers of IL-10 expressing lymphocytes were observed on a per islet basis (**Figure 6C**). We next asked if IL-10 protein was produced by analyzing IL-10 in the supernatant of cultured islets by ELISA. IL-10 was detectible in the supernatant of B6 islets at similar levels as NOD islets (**Figure 6D**).

**Figure 7:**
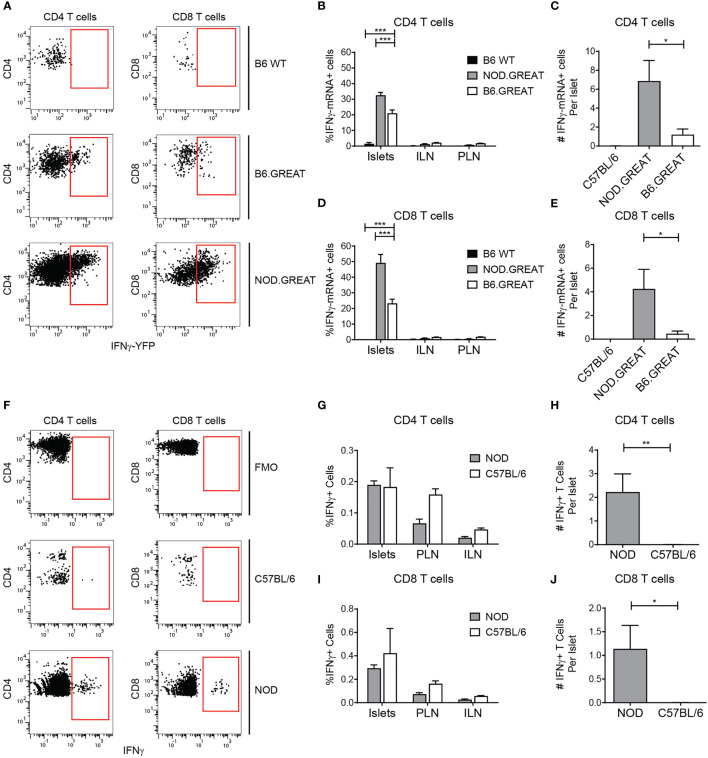
Islet T cells do not express IFN-γ in the steady state. Flow cytometry analysis of IFN-γ transcript in the GREAT IFN-γ transcript reporter **(A–E)** and IFN-γ protein expression by intra-cellular cytokine staining **(F–J)**. **(A)** Representative YFP expression in the GREAT IFN-γ transcript reporter in islets. **(B, D)** Percent IFN-γ−YFP positive T cells. **(C, E)** Number of IFN-γ−YFP positive T cells per islet. **(F)** Representative IFN-γ protein staining in islets. **(G, I)** Percent IFN-γprotein positive T cells. **(H, J)** Number of IFN-γprotein positive T cells per islet. **(B–D)** n=3 B6 WT, 7 B6.GREAT, 4 NOD.GREAT from 3 independent experiments. **(G–I)** n=8 B6 WT, 5 NOD from 3 independent experiments. **(A–J)** Statistics: *p < 0.05, **p < 0.01, ***p < 0.001. Error bars: SEM. **(B, D, G, J)** Two-way ANOVA with Bonferroni post-test. **(C, E, H, I)** One-way ANOVA with Tukey’s multiple comparisons.

Tissue-resident T cells are primed to produce inflammatory cytokines (32, 42), and B6 T cells are biased towards a Th1 effector phenotype (43, 44). Thus, we analyzed islet T cell expression of IFN-γ mRNA using the transcriptional IFN-γ reporter B6.GREAT mouse (31, 32, 42). IFN-γ transcript expression was compared to the inflamed setting in NOD.GREAT islets. A subset of islet T cells in B6 and NOD mice expressed IFN-γ transcripts (**Figures 7A–E**). However, the number of cells per islet that expressed IFN-γ transcripts was significantly lower in B6 than NOD islets (**Figures 7C–E**). Because IFN-γ is post-transcriptionally regulated, we analyzed IFN-γ protein production. IFN-γ protein was largely absent in the T cells of B6 islets, while a small number of T cells produced IFN-γ protein in NOD islets (**Figures 7F–J**). From these data, we concluded that T cells in non-diabetic B6 islets are poised but do not actively produce IFN-γ. This suggests that non-diabetic B6 islet T cells and B cells have a regulatory phenotype, due to IL-10 expression and lack of IFN-γ production.

### Islet T Cells and B Cells Maintain a Regulatory Phenotype Under Metabolic Stress

**Figure 8:**
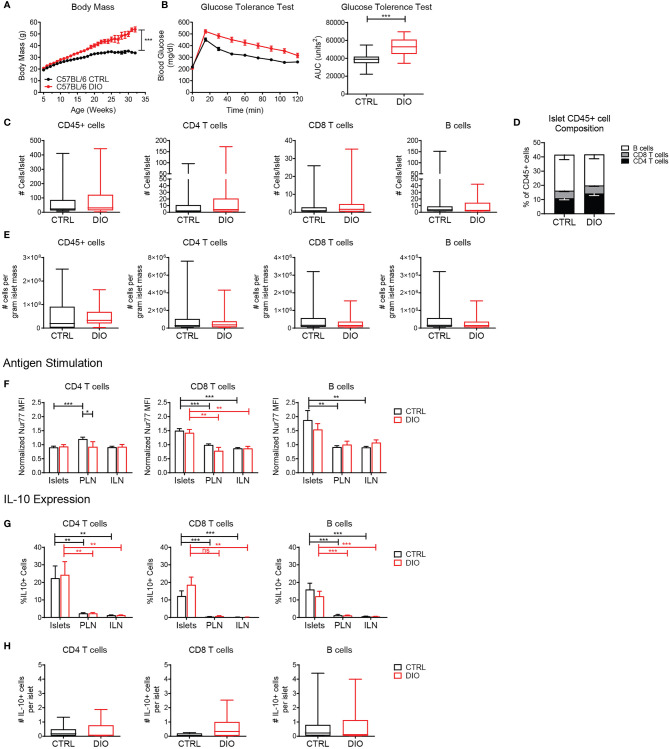
Islet T cells and B cells maintain a regulatory phenotype under metabolic stress in the diet-induced obesity model. Mice were maintained on a high fat diet (DIO) or control diet (CTRL). **(A)** Body weight. **(B)** Glucose tolerance test. Blood glucose levels and area under the curve (AUC) following glucose challenge. **(C)** Number of cells per islet in CTRL and DIO mice. **(D)** Percent of CD4 T cells, CD8 T cells and B cells of the islet CD45+ population. **(E)** Estimated number of cells per gram of islet tissue, based on 0.035mg islet weight per gram of mouse body weight. **(F)** Normalized Nur77-GFP reporter expression in CTRL and DIO B6.Nur77-GFP mice. **(G, H)** IL-10-GFP reporter expression in CTRL and DIO B6.TIGER mice. **(A–E)** n=32 control mice, 42 DIO mice from 12 independent experiments. **(F)** n=19 control mice, 8 DIO mice from 6 independent experiments. **(G, H)** n=10 control mice, 10 DIO mice from 3 independent experiments. **(A–H)** Statistics: *p < 0.05, **p < 0.01, ***p < 0.001, ns, not significant. Error bars: SEM. **(B, C)** Two-tailed student’s t test. **(A, F–H)** Two-way ANOVA with Bonferroni post-test. **(C, D)** Mann Whitney two tailed test.

To test the stability and function of the islet lymphocytes in pre-T2D, we used the DIO model (45). B6 mice were fed the DIO diet (60% of calories from fat) or a control diet (10% of calories from fat) from 4-6 weeks of age. At 16 weeks of age, a glucose tolerance test was performed. DIO mice gained weight and became glucose intolerant (**Figures 8A, B**).

When quantified by flow cytometry, we found that DIO islets contained similar numbers and percentages of T cells and B cells per islet compared to control fed mice (**Figures 8C–E**). B6 mice were previously determined to have approximately 0.035mg islet weight per gram of mouse body weight (46). Thus, we calculated an estimated number of cells per gram of islet tissue based on body weight (**Figure 8E**). DIO mice contained a similar number of cells per calculated gram of islet tissue compared to control-fed mice (**Figure 8E**) Consistent with our previous finding (**Figure 5**), islet CD8 T cells and B cells expressed elevated levels of the Nur77-GFP reporter indicating that they received local antigen stimulus. There was no difference between DIO mice and control-fed mice, indicating that local antigenic stimuli in the islets were unaltered under metabolic stress conditions (**Figure 8F**).

Since obesity and metabolic stress increase circulating pro-inflammatory cytokines (8, 9, 11), we asked if islet T cells and B cells maintained IL-10 expression under the inflammatory conditions in the DIO model (47). T cells and B cells maintained elevated expression of IL-10 in the islets in control-fed and DIO B6.TIGER mice as well as similar numbers of IL-10 expressing cells per islet (**Figures 8G, H**). This suggests that islet lymphocytes are stable populations that maintain their regulatory phenotype under metabolic stress.

## Discussion

In this study, we characterized islet lymphocyte populations in mouse islets under normal homeostasis and metabolic stress. Our results indicate that islet T cells and B cells receive local antigen stimulus and express a regulatory phenotype in normal and pre-T2D islets. These lymphocytes were localized in the islet parenchyma. They expressed the regulatory cytokine IL-10, and did not actively produce the pro-inflammatory cytokine IFN-γ. Islet T cell and B cell populations in pre-T2D islets maintained their regulatory phenotype and were present in similar numbers as control islets. These data suggest that islet T cells and B cells may be serving a protective role. T2D patient islets had an increase in CD4 T cells compared to non-diabetic donor islets. T cells in the T2D patient islets also showed an activated state based on CD44 expression in both CD4 and CD8 T cells, which was more similar to T1D islets than non-diabetic controls.

Understanding the function of immune cells in the islets is important for determining how they participate in diabetes pathogenesis. In non-diabetic mice, islet lymphocytes increase with age (18). Islet lymphocytes are also present in human islets and have a largely activated or memory phenotype (**Figure 3**) (14, 15, 48, 49). Though we did not observe a significant increase in CD8 T cell numbers in T2D islets compared to non-diabetic islets as others have shown, we did observe an increase in islet CD4 T cells. Notably, the activation state of the T cells from T2D islets was more similar to islet T cells derived from T1D donors than non-diabetic donors based on CD44 expression, suggesting an influence by the disease state (**Figure 3**). This work confirms the presence of lymphocytes within normal and T2D murine and human islets. We further show that these lymphocytes localize to the parenchyma of the islet, rather than in the vascular lumen.

Lymphocytes in the islets in both mouse and human were similar in number and frequency as other peripheral tissues such as the lung, liver, and kidney. For example, mouse islets contained approximately 1.6x10^6^ T cells per gram of tissue (**Figure 8E**), whereas T cell numbers in other peripheral tissues range from 2x10^4^ to 1.5x10^6^ per gram of tissue (50–55). Similarly, islet T cells comprise about 16% of the mouse and 5-20% of the human islet leukocyte compartment, whereas in other tissues lymphocytes comprised 15-55% of the leukocyte compartment (50–55).

In regards to phenotype, human islet T cells have a similar level of antigen experience and a memory phenotype as T cells in other peripheral tissues (52, 56). Their heterogenous expression of tissue resident markers is also similar to T cells in other tissues (54). In comparison to murine islet T cells, human islet T cells did not contain as much diversity in activation phenotype. The activated and memory state may allow islet T cells to respond rapidly to localized infections or tissue damage.

We found that murine islet T cell and B cell populations are comprised of multiple subsets. Surprisingly, a third of the islet T cells had a naïve phenotype. Naïve T cells have been identified in other non-lymphoid tissues in relatively small numbers and are thought to home to non-lymphoid tissues independently of chemokine receptor signaling (57). It has been suggested that naïve T cells may traffic through non-lymphoid tissues to maintain tolerance, but this mechanism is not well understood (57–59). Memory T cells and Tregs also populated the islets (**Figure 2**). The majority of non-regulatory islet B cell populations were naïve (**Figure 4**), which is consistent with identification of naïve B cells in other nonlymphoid tissues as part of their circulation and patrolling pathway (60). Importantly, the enrichment of regulatory B cell populations (Breg and transitional) in normal islets has not previously been identified (**Figure 4**) and represents a skewing of the islet lymphocyte compartment towards regulatory and homeostatic functions.

The skewing of the islet B cell compartment towards regulatory B cells may play a role in establishing this protective population in type 1 diabetes. Notably, Bregs have been identified in protecting islet from type 1 diabetes progression and have been implicated in protection following therapeutic intervention (61, 62). Furthermore, IL-10 expression by regulatory B cells may protect the islets from infiltration of B1a cells, which have been implicated in initiating autoimmune diabetes through interaction with neutrophils and pDCs (63–65). The distribution of islet B cells around the islet may allow for localized release of IL-10 to protect the islets (**Figure 1**).

Islet T cell localization in the parenchyma and the presence of memory T cells suggest that the activated/memory T cells may be Trms. Trms in mice and humans are present in non-lymphoid tissues including the pancreas (14, 66). Distinguishing Trms has proved challenging since expression of the tissue residence markers CD103 and CD69 may differ depending on the tissue (27, 67–69). We found that a subset of islet CD8 T cells express CD103 and CD69 (**Figure 2**). Trms express pro-inflammatory cytokine transcripts (32, 42). We show that islet CD8 T cells express IFN-γ transcripts, but do not actively produce IFN-γ protein. Similar to Trms identified in other studies, islet CD8 T cells express high levels of CD44 and receive local antigen stimulation by Nur77 expression, while the islet CD4 T cells did not show evidence of local antigen stimulation (30, 70, 71). This suggests that like Trms in other tissues, islet CD8 T cells may be poised to respond to infection rapidly (32, 72). CD4 Trms are less dependent on TCR signaling for maintaining tissue residence which is consistent with our finding that islet CD4 T cells have similar Nur77 levels as the matched population in the lymph nodes (**Figure 5**) (73, 74).

It is unknown what antigens stimulate the TCR and BCR signaling reported by Nur77. Notably, the Nur77 upregulation was modest, suggesting a response to low affinity antigen or low expressed antigen, which may in part explain why these lymphocytes do not lead to autoimmunity. Islet-antigen specific CD8 T cells, including T cells specific for preproinsulin and ZnT8, have recently been identified in human pancreas tissue of non-diabetic donors (75–77). These may represent some of the antigens that elicit Nur77 upregulation in normal islet T cells.

Our finding that islet T cells and B cells express IL-10, is significant since IL-10 can polarize macrophages to an anti-inflammatory M2-like state and suppress pro-inflammatory cytokine production including IFN-γ (78, 79). The local antigen stimulus may be important for expression of this regulatory cytokine. Under homeostatic conditions and in the DIO model, expression of IL-10 but not IFN-γ suggests that islet lymphocytes may maintain an anti-inflammatory environment. This role may include protecting beta cells in the islets from systemic inflammation, since beta cells can become stressed, dysfunctional or undergo apoptosis in response to proinflammatory cytokines (80, 81). In the context of T2D, prolonged elevated glucose levels can trigger beta cell stress and production of IL-1β by beta cells (13, 49, 82). The inflammatory environment can also initiate a shift in islet macrophages to an M1-like phenotype, further promoting inflammation and beta cell dysfunction and death (9). Notably, the DIO model does not develop hyperglycemia, which may in part be due to the IL-10 expressing islet lymphocytes (34, 45). Although we observed increased numbers of certain islet lymphocytes in human T2D patients, consistent with others’ analyses (14, 15), this was not observed in the DIO model. The difference between human T2D and the DIO model may be due to the differences in disease state, as the DIO model does not progress to overt diabetes (34, 45).

Islet T cells and B cells that are present under homeostatic conditions may also have implications for understanding autoimmune T1D. Our data suggest that the islet T cells and B cells play a regulatory role, which could delay initial autoimmune pathogenesis by autoreactive T cells in T1D. However, in T1D autoreactive T cells overcome these regulatory islet lymphocytes, resulting beta cell destruction. This may be in part because the islet T cells are poised to produce IFNγ. Thus, with localized inflammation, the islet T cells may convert to a pro-inflammatory state and promote autoimmune destruction.

Our data identify islet T cells and B cells as a regulatory population. This augments our understanding of how the immune system contributes to regulating the islet environment under homeostatic conditions and in pre-T2D. However, more investigation is needed to understand how islet lymphocytes are recruited to and maintained in the tissue, what antigens stimulate their TCR and BCR signaling, and what stimuli trigger IL-10 expression. Also, while naïve T cells and B cells have been found in other nonlymphoid tissues (57, 60), their function in the islets is unknown. Further study is also needed to characterize the protective role that islet T cells and B cells may play in T2D, and how anti-inflammatory treatments for T2D affect islet T cell and B cell populations.

## Materials and Methods

### Mice

C57BL/6 (000664), NOD/ShiLtJ (001976), B6.TIGER (008379), and B6.Nur77-GFP (016617) mice were obtained from The Jackson Laboratory and bred in-house. B6.GREAT mice were a gift from Dr. R. Lee Reinhardt and NOD.GREAT mice were a gift from Dr. Jeffrey Bluestone. All animal procedures were approved by the Institutional Animal Care and Use Committee at National Jewish Health and University of Colorado Anschutz Medical Campus. For additional information refer to electronic supplementary material.

### Diet-Induced Obesity Mice

DIO mice were generated in-house by feeding 4-6 week mice ad libitum a diet containing 60% of calories from fat (Research Diets, D12492) or a control diet containing 10% of calories from fat (Research Diets, D12450B) until euthanasia at 16-40 weeks of age. Weight and blood glucose levels were monitored weekly. At 16 weeks of age, mice were challenged by intraperitoneal injection of 2g glucose/kg in PBS and blood glucose levels were monitored every 15 minutes for 2 hours post injection. DIO mice were excluded from the study if weight gain was not within one standard deviation of the weight curve from The Jackson Laboratory (34).

### Islet Isolation

Islets were isolated as previously described (83–86). Following euthanasia, the pancreas was inflated *via* the common bile duct with ~3 ml of 0.8mg/ml Collagenase P (Roche) or 2.5 ml CIzyme RI (Vitacyte) and 10µg/ml Dnase I (Roche) in HBSS (Cellgro). The pancreas was removed and digested at 37°C. Islets were obtained by density centrifugation and hand-picked under a dissecting microscope. Single cell suspensions were made by digestion using 0.4 Wunsch Units/ml Collagenase D (Roche) and 250 µg/ml DNAse I (Roche) in HBSS (Cellgro) with 10% FBS (Hyclone) at 37°C for 30 min. Islets were dissociated in Cell Dissociation Buffer (Sigma) at 37°C for an additional 30 min.

### 2-Photon Imaging of Explanted Islets

Isolated islets stained with fluorescently labeled antibodies CD31 PE (eBioscience) and B220 FITC (Biolegend) or CD3 FITC (Biolegend) for 90 minutes on ice and then fixed with 2% paraformaldehyde. Islets were imaged at 810nm using an Olympus FV100MPE as described previously (83–85). 100 xy planes of 509 µm by 509 µm with a resolution of 0.994 µm/pixel and 1-um z-spacing were acquired. Image analysis was performed using Imaris (Bitplane) and MATLAB (Mathworks). Images were linearly unmixed, as previously described (83, 84).

### Flow Cytometry

Islets were isolated and digested as described above. Pancreatic (draining) or Inguinal (non-draining) lymph nodes were shredded using needles, and digested with 0.4 Wunsch Units/ml Collagenase D (Roche) and 250 µg/ml DNAse I (Roche) in HBSS (Cellgro) with 10% FBS (Hyclone) at 37°C for 30 min. Isolated islet and lymph node cells were stained with fluorescent antibodies for 30 minutes on ice (**Supplementary Material**).

For intracellular staining of IFN-γ, mice were treated i.v. with brefeldin A (Sigma) for 4 hours prior to harvest as previously described (85). Single cell suspensions of islets or lymph nodes were stained for surface markers. Following fixation and permeabilization using a FoxP3 Staining Kit (eBioscience), cells were stained with fluorescent antibodies against intracellular targets for 30 minutes on ice. FoxP3 staining was performed similarly.

Samples were analyzed on an LSR Fortessa (BD) or Aurora (Cytek). Analysis, compensation, and unmixing were performed in FlowJo (TreeStar). For antibodies, refer to electronic supplementary material.

### Islet Culture and Multiplex ELISA of Supernatants

Mice were treated i.v. with brefeldin A (Sigma) 4 hours prior to islet harvest (85). Isolated islets were placed in RPMI 1640 (Corning) with 10% FBS (Hyclone), 2.5% HEPES (Invitrogen) and 1% NEAA (Invitrogen) without brefeldin A for 24 hours. Supernatant was collected and analyzed for cytokine concentration by V-PLEX Proinflammatory Panel 1 Mouse Kit (Meso Scale Discovery).

### CyTOF Analysis From Human Pancreas Analysis Program Database

T2D and non-diabetic donors with no autoantibodies detected in the serum were selected from the Human Pancreas Analysis Program (HPAP) Database, consortia under Human Islet Research Network (RRID : SCR_014393) (37). The HPAP group isolated islets from cadaveric donors and approximately 10,000 IEQs were used for analysis by CyTOF. Islets were digested and cells were stained with cisplatin followed by fixation and staining with heavy metal barcoded antibodies against cell surface markers (16). CyTOF data from islets of donors was analyzed using FlowJo (TreeStar). For demographic information, refer to electronic supplementary material.

### Statistical Analysis

Statistical analysis was performed with Prism 8 software (GraphPad). Data are expressed as means with SEM. Specific statistical tests are noted in figure legends.

## Data Availability Statement

The raw data supporting the conclusions of this article will be made available by the authors, without undue reservation.

## Ethics Statement

The animal study was reviewed and approved by Institutional Animal Care and Use Committee at National Jewish Health and the University of Colorado Anschutz Medical Campus.

## Author Contributions

JCW, RSL, and RSF contributed to the conception and design of the study. All authors generated and analyzed data. JCW wrote the first draft of the manuscript. JCW, RSL, JGO-C, and RSF contributed to revising and editing the manuscript. All authors approved the submitted version. RSF acquired funding to support the project and supervised the project.

## Funding

This research was performed with the support of National Institutes of Health grant 5R21AI119942-02 (RSF), National Institute of Health grant 1R01DK111733-01 (RSF), Cancer Research Institute #AWD-112499 (to support RSL), National Institutes of Health grant 5T32AI007405-27 (to support JCW), and the University of Colorado Diabetes Research Center (National Institutes of Health grant P30-DK116073).

## Conflict of Interest

The authors declare that the research was conducted in the absence of any commercial or financial relationships that could be construed as a potential conflict of interest.

## Publisher’s Note

All claims expressed in this article are solely those of the authors and do not necessarily represent those of their affiliated organizations, or those of the publisher, the editors and the reviewers. Any product that may be evaluated in this article, or claim that may be made by its manufacturer, is not guaranteed or endorsed by the publisher.
