# Supplementary material for: Islet Lymphocytes Maintain a Stable Regulatory Phenotype Under Homeostatic Conditions and Metabolic Stress
Source: Front Immunol. 2022 Jan 25;13:814203. doi: 10.3389/fimmu.2022.814203 (PMC8821107; doi:10.3389/fimmu.2022.814203)
Supplement: Supplementary file 1 [file DataSheet_1.pdf]

Supplementary Material

Methods

Mice:

C57BL/6 (000664), NOD/ShiLtJ (001976), B6.TIGER (008379), and B6.Nur77-GFP (016617) mice were obtained from The Jackson Laboratory and bred in-house. B6.GREAT mice were a gift from Dr. Lee Reinhardt and NOD.GREAT mice were a gift from Dr. Jeffrey Bluestone. All animal procedures were approved by the Institutional Animal Care and Use Committee at National Jewish Health and University of Colorado Anschutz Medical Campus. Mice were housed in standard ventilated mice cages under specific pathogen free conditions and maintained at an ambient temperature of 22°C. Mice were provided bedding, nesting material and huts to improve quality and reduce fighting between male mice. Mice were placed in same-sex groups of 5 or less using a 12h dark/light cycles with ad libitum food and water. Both male and female mice between ages 12-40 weeks of age were analyzed for C57BL/6 background mice. Female NOD mice between the ages of 12-20 weeks of age were analyzed, as male NOD mice do not have a high penetrance of autoimmune diabetes with greater variability in progression to overt diabetes[1]. Mice were monitored daily for general health and well-being.

Diet-Induced Obesity (DIO) Mice

Diet-induced obesity (DIO) mice were generated in-house by feeding 4-6 week mice ad libitum a diet containing 60% of calories from fat (Research Diets, D12492) or a control diet containing 10% of calories from fat (Research Diets, D12450B) until euthanasia at 16-40 weeks of age. At or within 2 weeks of weaning, male littermates were randomized when assigned to a diet. Male mice were exclusively used for DIO studies as female mice have reduced capacity to gain appropriate weight on a high-fat diet. Weight and blood glucose levels were monitored weekly. At 16 weeks of age, mice were challenged by intraperitoneal injection of 2g glucose/kg in PBS and blood glucose levels were monitored every 15 minutes for 2 hours post injection. DIO mice were excluded from the study if weight gain was not within one standard deviation of the weight curve from The Jackson Laboratory[2].

Flow Cytometry:

Flow Cytometry Panels and Antibody Information:

Marker	Fluor	Vendor	Catalog Number	Dilution
Resident T cell Panel				
CD45	BUV395	BD	564279	(1:400)
CD90.2	FITC	Biolegend	105306	(1:200)
CD19	PE	Biolegend	115508	(1:200)
CD4	AlexaFluor594	Biolegend	100446	(1:200)
CD8a	BV650	Biolegend	100741	(1:200)
CD69	PE-Cy7	Biolegend	104511	(1:200)
CD11b	APC-Cy7	Biolegend	101225	(1:200)
CD103	BV785	Biolegend	121439	(1:200)
CD49d	AlexaFluor647	Biolegend	103613	(1:200)
CD44	BV510	Biolegend	103043	(1:200)
B cell subset Panel				
CD45	BUV395	BD	564279	(1:400)
CD19	BUV737	BD	612781	(1:200)

B220	BV421	Biolegend	103239	(1:200)
IgM	BV750	BD	747333	(1:200)
IgD	AlexaFluor700	Biolegend	405729	(1:200)
CD5	PerCP	Biolegend	100615	(1:200)
CD1d	PE	Biolegend	123510	(1:200)
CD138	BV605	BD	142515	(1:200)
CD43	APC	Biolegend	121214	(1:200)
CD80	FITC	eBioscience	11-0801-85	(1:200)
CD90.2	APC-Cy7	Biolegend	105328	(1:400)
Nur77-GFP Reporter Panel				
CD45	BUV395	BD	564279	(1:200)
CD90.2	PE-Cy7	Biolegend	105326	(1:200)
CD4	BV711	Biolegend	100447	(1:200)
CD8a	APC-eFluor780	Thermo-Fisher	47-0081-82	(1:200)
CD19	BUV737	BD	612781	(1:200)
CD62L	Pacific Blue	Biolegend	104424	(1:200)
CD44	BV510	Biolegend	103043	(1:200)
MHCII (I-A/I-E)	APC	eBioscience	17-5321-82	(1:600)
IL-10-GFP Reporter Panel				
CD45	BUV395	BD	564279	(1:200)
CD90.2	PE-Cy7	Biolegend	105326	(1:200)
CD4	BV711	Biolegend	100447	(1:200)
CD8a	APC-eFluor780	Thermo-Fisher	47-0081-82	(1:200)
CD11b	BV421	Biolegend	101236	(1:100)
CD19	PE	Biolegend	115508	(1:200)
B6.GREAT Reporter Panel				
CD45	BUV395	BD	564279	(1:200)
CD90.2	PE-Cy7	Biolegend	105326	(1:200)
CD4	BV711	Biolegend	100447	(1:200)
CD8	eFluor660	eBioscience	50-0081-82	(1:200)
CD11b	BV421	Biolegend	101236	(1:100)
CD19	PE	Biolegend	115508	(1:200)
Intracellular IFNg Panel				
CD45	BUV395	BD	564279	(1:200)
CD90.2	FITC	Biolegend	105306	(1:200)
CD4	BV711	Biolegend	100447	(1:200)
CD8	eFluor660	eBioscience	50-0081-82	(1:200)
CD11b	BV421	Biolegend	101236	(1:100)
CD19	PE	Biolegend	115508	(1:200)
IFNg	PE-Cy7	Biolegend	505825	(1:100)
FoxP3 Panel				

CD45	BUV395	BD	564279	(1:200)
CD90.2	FITC	Biolegend	105306	(1:200)
CD4	BV711	Biolegend	100447	(1:200)
CD8	PerCP-Cy5.5	Biolegend	100734	(1:200)
CD25	Biotin	Biolegend	102004	(1:200)
CD19	BV510	Biolegend	115545	(1:200)
FoxP3	PE	eBioscience	12-5773-82	(1:100)

CyTOF Analysis from Human Pancreas Analysis Program (HPAP) Database

Human CyTOF data was obtained from deidentified patient samples sourced from the Human Pancreas Analysis Program. Study size was based upon available number of patient data. Because the database was deidentified, potential sources of bias were removed.

Demographic information for donors used in the study:

Donor Number	Group	Age (years)	Sex	BMI (kg/m^2)	Race
HPAP-034	Control	13	Male	18.6	Caucasian
HPAP-035	Control	35	Male	26.91	Caucasian
HPAP-037	Control	35	Female	21.9	Caucasian
HPAP-040	Control	35	Male	23.98	Caucasian
HPAP-052	Control	27	Male	38.72	African American
HPAP-053	Control	58	Female	24.2	Caucasian
HPAP-054	Control	40	Female	30.85	Caucasian
HPAP-059	Control	35	Male	----	Caucasian
HPAP-051	T2D	43	Female	45.49	African American
HPAP-057	T2D	50	Female	30.49	Caucasian
HPAP-061	T2D	59	Female	38.27	African American
HPAP-020	T1D	14	Male	13.2	Caucasian
HPAP-021	T1D	13	Female	21.4	Caucasian
HPAP-023	T1D	17	Female	21.35	Caucasian
HPAP-028	T1D	4	Male	17.3	Caucasian
HPAP-032	T1D	10	Female	16.3	Caucasian
HPAP-055	T1D	24	Male	27.9	Caucasian
HPAP-064	T1D	24	Male	16.98	African American
HPAP-087	T1D	15	Female	19.3	Caucasian

References:

1. Leiter EH (1997) The NOD Mouse: A Model for Insulin-Dependent Diabetes Mellitus. Curr Protoc Immunol 24(1):15.9.1-15.9.23. https://doi.org/10.1002/0471142735.IM1509S24
2. Laboratory J PHENOTYPE INFORMATION FOR DIET-INDUCED OBESE C57BL/6J (380050). https://www.jax.org/jax-mice-and-services/strain-data-sheet-pages/phenotype-information-380050#
